# Protective vs. Therapeutic Effects of Mitochondria-Targeted Antioxidant MitoTEMPO on Rat Sciatic Nerve Crush Injury: A Comprehensive Electrophysiological Analysis

**DOI:** 10.3390/biomedicines11123306

**Published:** 2023-12-14

**Authors:** Murat Cenk Celen, Ahmet Akkoca, Seckin Tuncer, Nizamettin Dalkilic, Barkin Ilhan

**Affiliations:** 1 Department of Biophysics, Faculty of Medicine, Ankara Medipol University, 06570 Ankara, Türkiye; 2 Department of Occupational Health and Safety, Taskent Vocational School, Selcuk University, 42960 Konya, Türkiye; 3 Department of Biophysics, Faculty of Medicine, Eskisehir Osmangazi University, 26040 Eskisehir, Türkiye; 4 Department of Biophysics, Faculty of Medicine, Baskent University, 06490 Ankara, Türkiye; 5 Department of Biophysics, Meram School of Medicine, Necmettin Erbakan University, 42090 Konya, Türkiye

**Keywords:** sciatic nerve, crush injury, MitoTEMPO, nerve conduction, therapeutic intervention

## Abstract

Protective vs. Therapeutic Effects of Mitochondria-Targeted Antioxidant MitoTEMPO on Rat Sciatic Nerve Crush Injury: A Comprehensive Electrophysiological Analysis. Peripheral nerve injuries often result in long-lasting functional deficits, prompting the need for effective interventions. MitoTEMPO (2-(2,2,6,6-tetramethylpiperidin-1-oxyl-4-ylamino)-2-oxoethyl) triphenylphosphonium chloride) is a mitochondria-targeted antioxidant that has shown protective and therapeutic effects against pathologies associated with reactive oxygen species. This study explores the utilization of MitoTEMPO as a therapeutic and protective agent for sciatic nerve crush injuries. By employing advanced mathematical approaches, the study seeks to comprehensively analyze nerve conduction parameters, nerve excitability, and the distribution of nerve conduction velocities to gauge the potential. Forty Wistar-Albino rats were randomly divided into following groups: (I) SHAM—animals subjected to sham operation and treated intraperitoneally (i.p.) with vehicle (bidistilled water) for 14 days; (II) CI (crush injury)—animals subjected to CI and treated with vehicle 14 days; (III) MiP—animals subjected to 7 days i.p. MitoTEMPO treatment before CI (0.7 mg/kg/day dissolved in vehicle) and, only vehicle for 7 days after CI, protective MitoTEMPO; and (IV) MiT—animals i.p. treated with only vehicle for 7 days before CI and 7 days with MitoTEMPO (0.7 mg/kg/day dissolved in vehicle) after CI, therapeutic MitoTEMPO. Nerve excitability parameters were measured, including rheobase and chronaxie, along with compound action potential (CAP) recordings. Advanced mathematical analyses were applied to CAP recordings to determine nerve conduction velocities and distribution patterns. The study revealed significant differences in nerve excitability parameters between groups. Nerve conduction velocity was notably reduced in the MiP and CI groups, whereas CAP area values were diminished in the MiP and CI groups compared to the MiT group. Furthermore, CAP velocity was lower in the MiP and CI groups, and maximum depolarization values were markedly lower in the MiP and CI groups compared to the SHAM group. The distribution of nerve conduction velocities indicated alterations in the composition of nerve fiber groups following crush injuries. In conclusion, postoperative MitoTEMPO administration demonstrated promising results in mitigating the detrimental effects of nerve crush injuries.

## 1. Introduction

Peripheral nerves comprise axons with varying conduction velocities, enclosed within a sheath. Activation of a single nerve results in the linear summation of action potentials originating from individual nerve fibers, forming the compound action potential (CAP). The CAP, when recorded from the nerve trunk, provides details about the quantity of active fibers and the velocities at which their action potentials propagate [1]. Compromised peripheral nerves due to injury can lead to transient or lifelong neuronal dysfunction, consequently contributing to subsequent socioeconomic challenges [2]. The nerve conduction parameters commonly used in the diagnosis of peripheral nerve disorders today actually possess the potential to provide more information with the help of advanced mathematical approaches [3]. Following the discovery of this potential, numerous numerical methods have been developed over the years to uncover such information [1,4,5].

Crush injuries, a frequently encountered category of peripheral nerve damage, have been extensively explored through experimental models. The rationale behind the frequent occurrence of such studies is their ability to mimic mechanical damage commonly observed in real-life scenarios [6,7,8]. The seminal work by Beer et al. in 2001 has established a foundational reference for inducing crush injuries, remaining influential over the last two decades. This study not only outlined the methodology for inflicting such injuries but also underscored the significance of standardization [9]. Subsequent investigations by Renno et al. have delved into diverse methodologies to examine the impact of various therapeutic agents on the recovery trajectory post-injury [10,11,12]. Meanwhile, Varejao et al. directed their efforts toward enhancing standardization during the induction of injuries [13]. While crush injury is physical damage, oxidative stress is recognized as a primary contributor to neural damage following injury, exerting an adverse impact on the recovery of nerve function after peripheral nerve injury.

Mitochondria are an important source of ROS (reactive oxygen species) within most mammalian cells [14]. The distribution of mitochondria is non-uniform across different parts of the neuron; for instance, Ranvier nodes exhibit a higher density of mitochondria compared to the remaining axon [15]. Additionally, this distribution varies between injured and non-injured axons. In the case of an injured axon, the mitochondrial count is twice as high as that in a non-injured axon [16]. This discrepancy arises from the essential role of mitochondria-produced ATP in facilitating axonal regeneration.

Antioxidants form a group of molecules capable of neutralizing the oxidative damage caused by free radical species and are naturally produced by the body. In peripheral nerve crush injuries, antioxidants such as alpha-lipoic acid, curcumin, isoquercitrin, sesame oil, and ginger oil have beneficial effects on axon regeneration [17,18,19,20]. Mitochondria, as the primary source of oxidative stress within cells, are a central target of antioxidants, leading to the development of mitochondria-specific antioxidants using various chemical compounds [21,22,23,24]. One such mitochondria-specific antioxidant is MitoTEMPO (2-(2,2,6,6-tetramethylpiperidin-1-oxyl-4-ylamino)-2-oxoethyl) triphenylphosphonium chloride) [25]. In the case of MitoTEMPO, triphenylphosphonium is the mitochondrial-targeting moiety, which selectively accumulates in mitochondria due to its unusually high membrane potential, whereas the rest of the molecule is the functional moiety [26].

This study aims to explore the effects of MitoTEMPO, employed as a protective or therapeutic agent in rat sciatic nerve crush injury model, which emulates a prevalent form of mechanical trauma experienced in real-life scenarios. The study seeks to evaluate how the utilization of MitoTEMPO impacts the recovery of sciatic nerve conduction and excitability parameters. 

## 2. Materials and Methods

### 2.1. Animal Preparation

Forty male Wistar-Albino rats were used in these experiments, and their weights were between 280–300 g. Rats were housed at cages three or four together and at controlled temperature 22 ± 2 °C and humidity on 12/12-light/dark cycle. All groups had free access to standard food ad libitum. Animals were weighed weekly to monitor potential weight fluctuations. This study was approved by Necmettin Erbakan University Experimental Medical Application and Research Center, Local Ethics Committee for Animal Experiments (Approval no: 2020-40). All animal experiments were performed in compliance with the ARRIVE guidelines.

**Figure 1:**
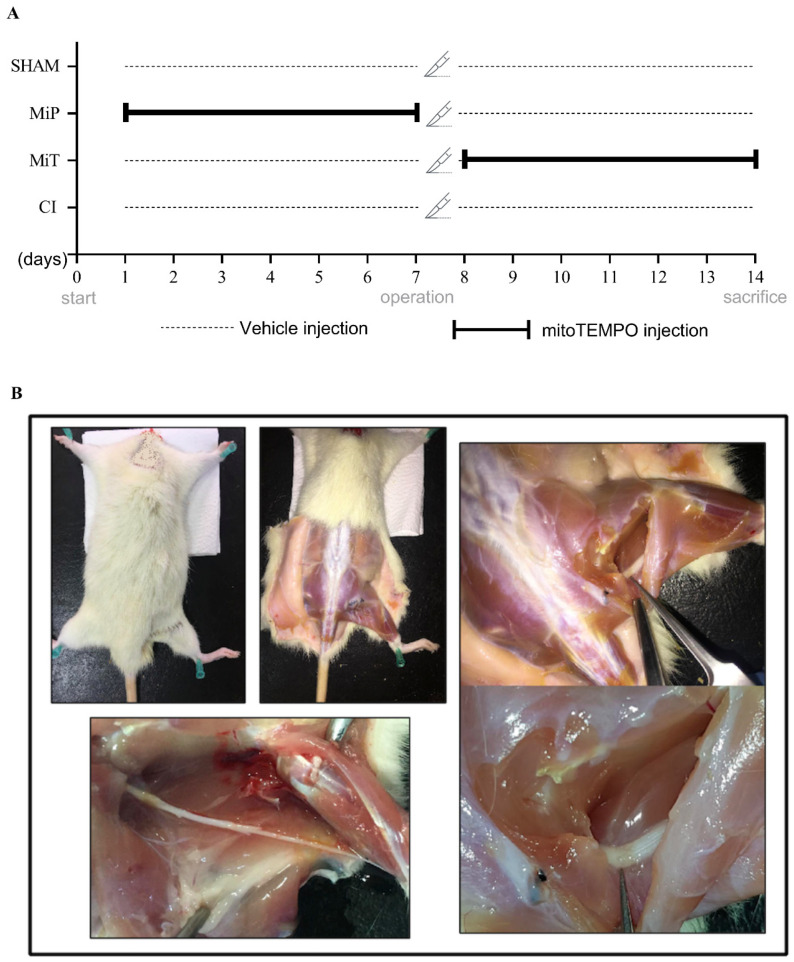
(**A**) Representation of experimental plan and applications. SHAM: Sham group; MiP: MitoTEMPO protective group; MiT: MitoTEMPO therapeutic group; CI: Crush injury group. (**B**) The upper left photo illustrates the dissection of the sciatic nerve from a rat. In this image, the rat’s skin has been removed, revealing the underlying musculature. In the photo on the right, the sciatic nerve is exposed, and the injury site is indicated with forceps. The lower left photo depicts the sciatic nerve at the moment just before removal for nerve conduction studies, capturing the extent of the injury.

Forty Wistar-Albino rats were randomly divided into following groups: (I) SHAM—animals subjected to sham operation and treated intraperitoneally (i.p.) with vehicle (bidistilled water) for 14 days; (II) CI (crush injury)—animals subjected to CI and treated with vehicle 14 days; (III) MiP—animals subjected to 7 days i.p. MitoTEMPO (Cat No. 1334850-99-5; Sigma-Aldrich, Darmstadt, Germany) treatment before CI (0.7 mg/kg/day dissolved in vehicle) and, only vehicle for 7 days after CI, protective MitoTEMPO; and (IV) MiT—animals i.p. treated with only vehicle for 7 days before CI and 7 days with MitoTEMPO (0.7 mg/kg/day dissolved in vehicle) after CI, therapeutic MitoTEMPO (Figure 1) [27].

There are studies from other research groups in various pathologies, as well as from our laboratory in ischemia-reperfusion injury, demonstrating therapeutic and/or protective effects of MitoTEMPO when applied at a dose of 0.7 mg/kg/day [28,29,30,31]. Therefore, this dose has been chosen in the current study.

Before the surgical procedures, rats were anesthetized with 100–200 mL/min O^2^ + 3–4% sevoflurone with the help of an inhalation device (Beyza Medikal Sevo-Inh, Ankara, Türkiye). The right thigh region was prepared for aseptic surgery and the animal was fixed by laying prone on a rectal temperature-controlled tray (MAY RTC 9404-A Animal Rectal Temperature Controller, Ankara, Türkiye). Linear skin incision (1–2 cm) was made on the caudo-lateral surface of the animal’s right thigh, and blunt dissection was performed to separate the biceps femoris and semitendinosus muscle to expose the sciatic nerve. Kreb’s solution (in mmol per liter: NaCl 119, KCl 4.8, CaCl_2_ 1.8, MgSO_4_ 1.2, KH_2_PO_4_ 1.2, NaHCO_3_ 20, and glucose 10, pH 7.4, and gassed with a mixture of 95% O_2_ and 5% CO_2_) at a constant rate of 5 mL per min at constant temperature (37 ± 0.5 °C) was dropped frequently, and the fluid loss of the tissues was prevented. Except from SHAM group, a crush injury was applied to the nerve with the help of curved hemostatic forceps (jaw width 3 mm). The force used for compression was standardized for 30 seconds at the second lock position of the hemostatic forceps (Hartmann Surgical Ins., Heidenheim, Germany) [9]. The location of the crush injury was determined as the middle part of the sciatic nerve that descends from the thigh area before bifurcating into the tibial and peroneal nerves. Muscles and skin were sewn in a standard way. Postoperatively, all animals were kept in individual boxes, and a single broad spectrum antibiotic gentamicin (32 mg/kg/day) and tramadol hydrochloride (4 mg/kg/day) were administered intramuscularly.

### 2.2. Nerve Dissection and Experimental Setup

At the end of 14 days of experiment, rats were decapitated, and they were quickly taken to the dissection table. Then, incisions were performed on the operated hind paws of rats, and sciatic nerves were dissected (Figure 1). Kindly isolated sciatic nerves were transferred into a heat-jacketed recording chamber, which was perfused with fresh modified Krebs solution. Two main electrophysiological parameters, compound action potential (CAP), and rheobase-chronaxie recordings were performed. Data visualization and calculations were performed as Werner Irnich (2010) indicated in detail on his previous study [32]. CAP with the stimulus of square-wave pulses had 0.1 ms duration and 1 Hz frequency, and, at the level of the SHAM group, the supramaximal value was 8 volts by using computer-controlled stimulator (Grass S88, Grass Instruments, Quincy, MA, USA). For the rheobase-chronaxie records between 0.01 and 0.1 ms duration, 1 Hz stimulus pulses were applied, and the triggering values (millivolts) of visible CAPs were recorded. After stimulus, which was applied from the proximal of the nerve bundle, CAP signals were recorded from the distal end by using a suction electrode. Amplified (Grass CP511, Grass Instruments, Quincy, MA, USA) CAPs were digitized (Advantech PCL1710 A/D converter, Taipei, Taiwan) at a 50 Hz sampling rate and acquired with the open-source software RETICAP http://icon.unrlabs.org/projects/reticap (accessed on 12 December 2023), which was developed in our laboratory.

### 2.3. Data Analysis

After getting the strength–duration curve, which is used in the examination of Rheobase–Chronaxie records, a mathematical formula is employed for its analysis. The mathematical expression of this curve as follows:

$$V=\left(V_{0}-P\right)\cdot e^{\left(-\tau /t\right)}+P$$

where V_0_: amplitude at theoretical moment when the stimulus duration is zero; P: required stimulus intensity when the stimulus duration is considered sufficiently long; and τ: the constant rate of descent for the curve.

To investigate the neural function for experimental groups, advanced mathematical procedures were conducted on all CAP recordings. Areas (mV·ms) under the CAP and the maximum depolarization (MD) value (mV) are proportional to the number of excited nerve fibers in that nerve. We have calculated the conduction velocity of the fastest fibers (V_cap_). The maximum and minimum time derivatives of CAPs (dV/dt_max_ and dV/dt_min_) relate the maximum and minimum rates of change in the rising and falling phases of the CAP with time and can be used as an index of the conduction activity of nerve fibers in a bundle. A suitable mathematical model that is the non-invasive method is needed to estimate conduction velocity distributions (CVDs) from CAPs recorded at certain distances from the stimulus site. To obtain the individual nerve conduction group activity, we estimate the nerve conduction velocity histogram by using the model that depends on Cummins et al., which we enhanced beforehand [33,34].

The basic principle of the model based on the statements of CAP can be expressed as:

$$\mathrm{CAP}\left(t\right)=\sum_{i=1}^{N}w_{i}\cdot f_{i}\cdot \left(t-{\;\tau }_{i}\right)$$

where CAP(t): the observed compound action potential as a function of time; N is the number of fiber classes; w_i_ is the amplitude weighting coefficients for class I; and f_i_(t) is the single-fiber action potential in class i. The weighting coefficients (w_i_) are general parameters to account for all influence on the contribution of each fiber class to the observed CAP. To estimate the individual activities of nerve fiber groups from CAPs, the CVDs for all nerves of the SHAM, MiP, MiT, and CI groups were calculated. The CVD histogram is divided into three subgroups, Slow (0–35 m/s), Medium (36–61 m/s), and Fast (62–88 m/s), as the visually augmented effect of MitoTEMPO on nerve conduction velocity can be helpful for ease of interpretation.

### 2.4. Statistics

Normality of data was tested with histograms and Kolmogorov–Smirnov test for the continuous variables. To compare the groups, one-way analysis of variance (ANOVA) followed by Duncan post hoc test were used for multiple comparisons when analysis of variance indicated significant results. All data were expressed as mean ± SEM (standard error of means) for each group, and significance was assumed at *p* < 0.05.

## 3. Results

### 3.1. General Findings

The weight fluctuations of the 40 animals in the 4 different groups included in the experiment were monitored. These changes were measured at the initial stage, at the end of the first week, and at the end of the second week. In a total of three measurements, no significant changes in animal weights were observed. The approximate weights at which the animals were sacrificed ranged between 320–340 g.

### 3.2. Nerve Excitability Parameters

**Figure 2:**
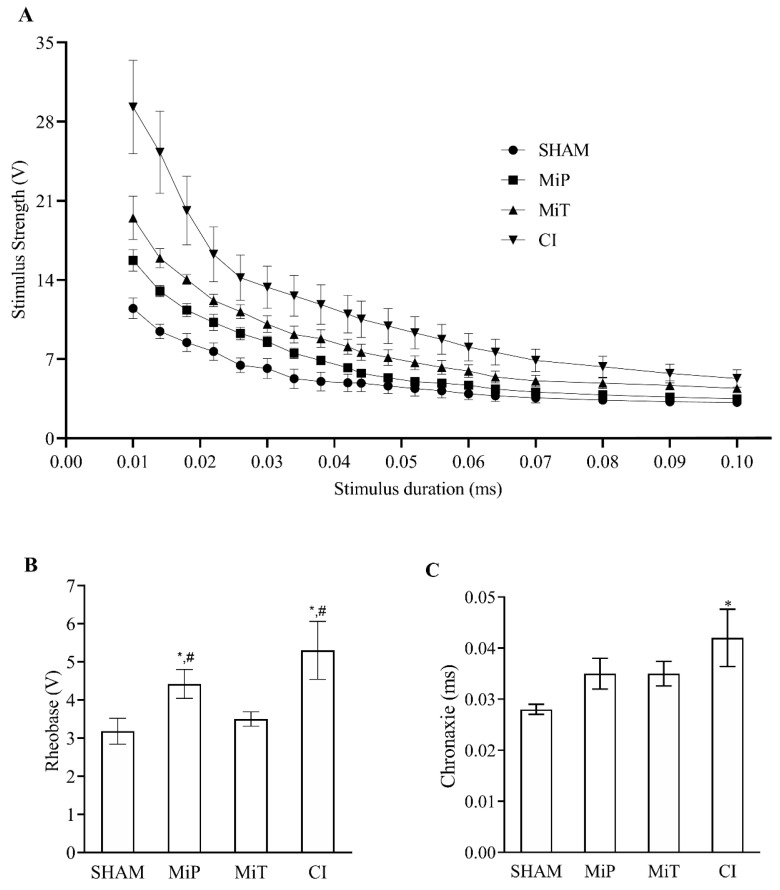
(**A**) Line graph of stimulus strength–duration values for four groups with SEM. (**B**) Rheobase values for four groups with bar graph (values are given as mean ± SEM. * indicates significant difference from SHAM group, and # indicates significant difference from MiT group *p* < 0.05). (**C**) Chronaxie values for four groups with bar graph (values are given as mean ± SEM. * indicates significant difference from SHAM group) SHAM: Sham group; MiP: MitoTEMPO protective group; MiT: MitoTEMPO therapeutic group; CI: Crush injury group.

Nerve excitability recordings were collected from the SHAM (n = 8), MiP (n = 6), MiT (n = 8), and CI (n = 7) groups. The mean values of the parameters τ, P, and regression coefficient (R^2^), calculated from the strength–duration curves shown in Figure 2A, along with their SEM, are provided in Table 1.

When the values obtained from Figure 2A and Table 1 are examined together, it can be observed that the strength–duration curves shift upwards for Sham, MiT, MiP, and CI, respectively. Additionally, as seen in the table, the P value has significantly changed for each group (*p* < 0.05). On the other hand, Rheobase (Figure 2B) and Chronaxie (Figure 2C) values calculated from each curve are also presented. From Figure 2B, it is evident that the Rheobase values of the MiP and CI groups are significantly higher than both the SHAM and MiT groups (*p* < 0.05). In Figure 2C, only the CI group shows a significantly higher chronaxie value compared to the SHAM group (*p* < 0.05).

### 3.3. Compound Action Potential Parameters

**Figure 3:**
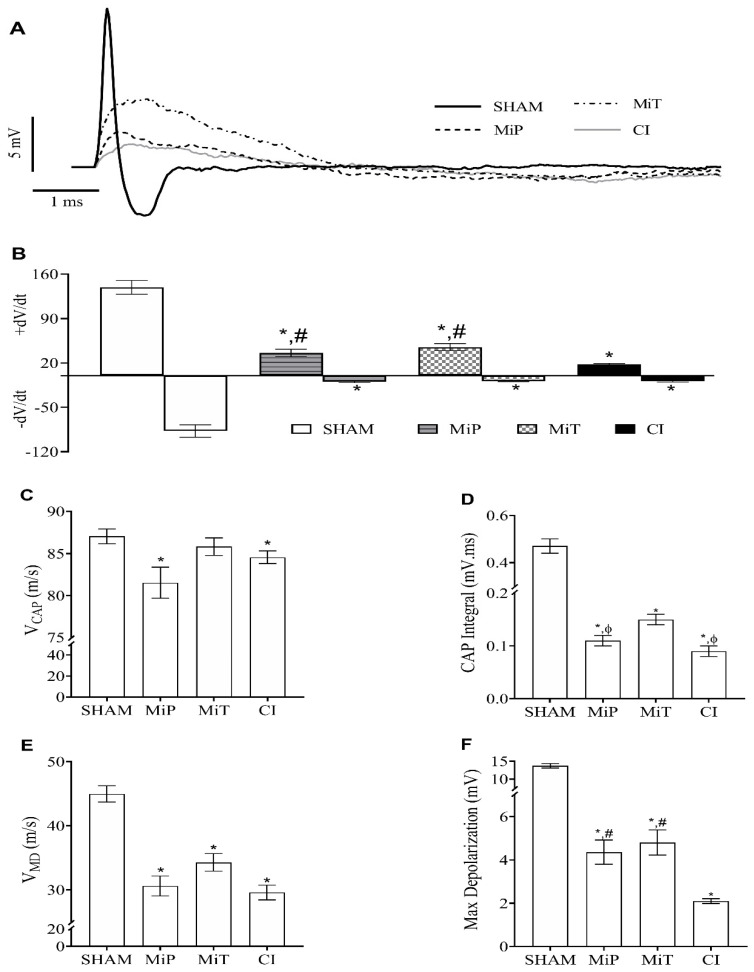
(**A**) Representative CAP (compound action potential) samples recorded from rat sciatic nerves of experimental groups, stimulus artifacts are not shown, and traces start from the end of the artifacts. (**B**) Positive and negative derivatives of CAP recordings for all groups. (**C**) Conduction velocity of CAPs (V_CAP_) for all groups (represents fastest ones). (**D**) The calculated area of CAP traces which is also called as CAP Integral. (**E**) Mean velocity values of maximum depolarization for the groups (V_MD_). (**F**) Mean values for maximum depolarization (MD). (Values are given as mean ± SEM. * indicates significant difference from SHAM group. ϕ indicates significant difference from MiT group, and # indicates significant difference from CI group in the graphs with *p* value < 0.05). SHAM: Sham group; MiP: MitoTEMPO protective group; MiT: MitoTEMPO therapeutic group; CI: Crush injury group.

CAP recordings were collected from SHAM (n = 10), MiP (n = 9), MiT (n = 9), and CI (n = 10) groups. An example record for all experimental groups is presented on the same axis in Figure 3A. The mean values of derivative maximum and derivative minimum for all groups are presented as a column graph on the same axis in Figure 3B. When the calculated minimum derivative values ((dV/dt)_min_) for the MiP, MiT, and CI groups are compared with the SHAM group value, they were significantly lower than the SHAM group. As for the ((dV/dt)_max_) value, the same groups are significantly lower than the SHAM group; however, the MiP and MiT groups are significantly higher than the CI group.

As seen in Figure 3C, when CAP velocity values are compared, it is observed that the MiP (81.35 ± 1.85 m/s) and CI (84.57 ± 0.76 m/s) groups are significantly (*p* < 0.05) slower than the SHAM (87.03 ± 0.88 m/s) group. In Figure 3D, the initial finding in CAP area values is that there is an approximate 50–60% reduction in groups subjected to crush injury, leading to a significant decrease (*p* < 0.05) in CAP area values compared to the SHAM group (0.47 ± 0.03 mV.ms). Another significant observation is that when the area of the CAP for the MiT group (0.150 ± 0.010 mV.ms) is compared to the area of CAPs for the MiP (0.110 ± 0.010 mV.ms) and CI (0.09 ± 0.01 mV.ms) groups, there are significant reductions in the CAP areas of these two groups compared to the MiT group. However, there is no significant difference in CAP area values between the MiP and CI groups.

The mean V_MD_ values for all groups are provided as a column graph in Figure 3E. The mean V_MD_ value for the SHAM group was calculated as 44.97 ± 1.27 m/s. When compared to the SHAM group value, there were significant differences (*p* < 0.05) for each group in terms of V_MD_ values (MiP; 30.61 ± 1.56 m/s, MiT; 33.13 ± 1.80 m/s, CI; 29.60 ± 1.15 m/s). On the other hand, when the MiP, MiT, and CI groups were compared among themselves, no significant differences were found. The mean value of maximum depolarization for the SHAM group was measured as 13.73 ± 0.63 mV. Significant decreases are observed in the mean maximum depolarization values for the other groups. These values are as follows: 2.10 ± 0.11 mV for the CI group, 4.36 ± 0.56 mV for the MiP group, and 4.81 ± 0.58 mV for the MiT group. When these mean maximum depolarization values of the groups were compared with the mean value of the SHAM group, significant differences were found for all groups. Additionally, the maximum depolarization value of the CI group is significantly lower than both the MiP and MiT values.

### 3.4. Conduction Velocity Distribution Parameters

**Figure 4:**
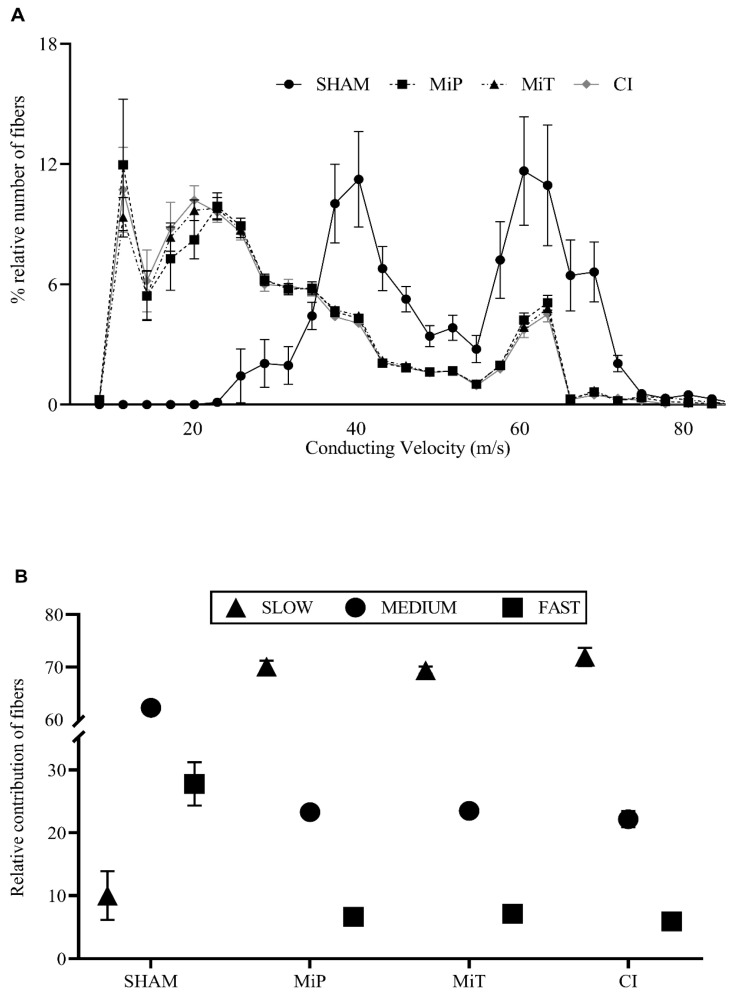
(**A**) Distribution of relative number of fibers percentage versus conducting velocity for all groups. (**B**) Relative contributions of major conduction velocity groups (slow. <35 m/s; medium. 36–61 m/s; and fast. 62–88 m/s) to CAPs recorded from experimental groups. SHAM: Sham group; MiP: MitoTEMPO protective group; MiT: MitoTEMPO therapeutic group; CI: Crush injury group.

The percentage values of subgroups within the four experimental groups (SHAM, MiP, MiT, and CI) are presented on the same axis in Figure 4A, accompanied by their SEMs for comparison. In Figure 4A, the nerve conduction velocity distribution for the SHAM group ranges from approximately 20 m/s to 80 m/s. In contrast, the other groups’ distribution shifts to the left, with velocities ranging from 2 m/s to 80 m/s showing a leftward shift compared to the SHAM group. The histograms represent the percentage-relative number of fiber groups within a bundle. It is important to consider these data in conjunction with other results. Such a shift is actually interpreted considering the alteration of contributing fibers. Another widely accepted method to make the results easily understandable is to categorize the velocity groups as slow (0–35 m/s), moderate (36–61 m/s), and fast (62–88 m/s). The visualization obtained based on this categorization is important to demonstrate the shifting among the generalized velocity groups across the experimental groups (Figure 4B).

## 4. Discussion

In our study, we aimed to investigate the effects of MitoTEMPO, a mitochondria-specific antioxidant, on sciatic nerve crush injuries in a rat model. Mitochondria, essential cellular organelles, have a vital function in upholding cellular homeostasis, particularly within neurons. Due to their distinct metabolic needs, different segments of neurons exhibit a non-uniform distribution of mitochondria. Regions with elevated ATP demands, such as presynaptic and postsynaptic terminals, active growth cones or axonal branches, and Ranvier nodes, harbor a greater density of mitochondria compared to other cellular regions [15]. In the context of nerve injuries, such as crush injuries, the density of axonal mitochondria may increase as a compensatory mechanism because Mitochondrial ATP production supports axon regeneration [16].

Mitochondria are a major source of reactive oxygen species (ROS) within cells, and excessive ROS production can lead to oxidative damage, affecting mitochondrial function [14].

Recent studies highlight the use of mitochondria-targeted antioxidants, such as MitoTEMPO, which typically utilize triphenylphosphonium for mitochondria targeting. The functional moiety of MitoTEMPO, acting as a trap for ROS, is delivered to mitochondria, where it can scavenge mitochondrial ROS and mitigate oxidative damage [26]. Furthermore, studies have shown that MitoTEMPO can modulate mitochondrial function and structure. In spinal cord injury models, MitoTEMPO has been demonstrated to remove mitochondrial ROS effectively, leading to enhanced spinal cord angiogenesis and functional recovery [35]. The therapeutic efficacy of MitoTEMPO in mitigating mitochondrial oxidative stress aligns with our findings in the context of nerve crush injuries.

Electrophysiological recordings in our study have shown significantly increased rheobase values in CI and MiP groups after crush injury (Figure 2B). Toraman et al. also studied a crush injury model on facial nerve of rats and recorded increased nerve excitability thresholds 3 weeks after injury [36]. The fact that the rheobase of the MiT and SHAM groups have no significant differences indicates that the therapeutic application of MitoTEMPO has yielded a positive outcome in terms of excitability. In their study, Tuncer et al. recorded compound action potentials in the streptozocin-induced diabetic rats’ sciatic nerves, even in the second and fourth weeks. They demonstrated that the shift in compound action potential, observed in different diabetic periods, mainly originated from fast-conducting nerve fibers [37]. In such cases, derivative values are also affected. The change in the V_CAP_ graph (Figure 3C) reveals a slowing down of the fastest fibers for the MiP and CI groups. This observation is substantiated by data from Ramli et al., who conducted a study involving 72 rats, inducing crush injury on the sciatic nerve, and conducting a comprehensive morphological and morphometric analysis of the affected nerves. Their findings demonstrated a greater impact of the injury on myelinated nerves compared to others. Our electrophysiological recordings align with and complement their histological analysis [38]. On the other hand, the MiT group has diverged from the other crush injury groups in terms of this parameter. The unit mV.ms shows that the CAP integral is significant, as it informs us about the magnitude of fiber involvement. Based on the data presented in Figure 3D, the MiT group contributes with more cells to CAP recordings than the MiP and CI groups. These results suggest that MitoTEMPO administered post-injury, as demonstrated in other studies, exerts an antioxidant effect within the mitochondria, supporting the necessary ATP synthesis for axonal regeneration [39]. Consequently, this facilitates a greater contribution of nerve axons to compound action potential, as indicated. As depicted in Figure 3E, the V_MD_ value of the SHAM group is significantly higher than the other three groups. This is another inference, indicating the decrease in the contribution of fast-conducting fiber groups and the shift of weight towards intermediate or slow-conducting nerve fibers. When maximum depolarization (Figure 3F) data are considered in conjunction with the integral data, it can be concluded that the application of MitoTEMPO, even as a protective measure, contributes to rapid fibers. In Figure 4, in groups exposed to crush injury, the proportional contribution of fast fibers has evidently decreased. Thus, considering the area (Figure 3D) in nerves exposed to crush injury, both the total number of contributing nerve fibers and the characteristics of these contributing fibers have changed. In groups exposed to MitoTEMPO (MiP and MiT), the total number of fibers diminished less than that of CI. However, the relative contribution shift in Figure 4A is similar in the MiT, MiP, and CI groups. This indicates that exposure to MitoTEMPO protects and recovers fast-conducting fibers more.

There are several limitations to this study. These include the absence of morphological aspects (such as myelin thickness, axonal thickness, number of myelinated axons, etc.), molecular analysis, assessment of sensory and motor functions, absence of dosage variation, and the utilization of a sole administration route for MitoTEMPO. To avoid unnecessary use of laboratory animals, in this study, we preferred the use of a single dose, which has already shown positive results in other studies, instead of trying different doses and administration routes.

## 5. Conclusions

This comprehensive study sheds light on the potential therapeutic and protective effects of MitoTEMPO in the context of sciatic nerve crush injuries in a rat model. The findings highlight significant alterations in nerve excitability parameters, nerve conduction velocities, and the distribution of nerve conduction velocities among different fiber groups following crush injuries. Notably, postoperative MitoTEMPO administration demonstrated promising results in mitigating the detrimental effects of nerve crush injuries, although not fully restoring them to baseline levels. These results emphasize the importance of considering mitochondria-specific antioxidants as potential interventions in nerve injury scenarios.

## Figures and Tables

**Table 1:** Calculated rate constant (τ) (at t = 0.01 ms), plateau (P), and regression coefficients (R^2^) for all groups (SHAM: Sham group; MiP: MitoTEMPO protective group; MiT: MitoTEMPO therapeutic group; CI: Crush injury group).

	SHAM	MiP	MiT	CI
**τ (** **ms)**	0.56 ± 0.11	0.76 ± 0.11	0.46 ± 0.07	0.66 ± 0.06
**P (V)**	3.18 ± 0.34	4.42 ± 0.38	3.50 ± 0.19	5.30 ± 0.76
**R^2^**	0.9943	0.9927	0.9855	0.9914

## Data Availability

All data generated or analyzed during this study are available upon request.
